# Mental Health, Greenness, and Nature Related Behaviors in the Adult Population of Stockholm County during COVID-19-Related Restrictions

**DOI:** 10.3390/ijerph18063303

**Published:** 2021-03-23

**Authors:** Mare Lõhmus, Cecilia U. D. Stenfors, Tomas Lind, André Lauber, Antonios Georgelis

**Affiliations:** 1 Center for Occupational and Environmental Medicine, Region Stockholm, 113 65 Stockholm, Sweden; tomas.lind@sll.se (T.L.); andre.lauber@sll.se (A.L.); antonios.georgelis@sll.se (A.G.); 2 Institute of Environmental Medicine, Karolinska Institute, Box 210, 171 77 Stockholm, Sweden; 3 Department of Psychology, Stockholm University, 106 91 Stockholm, Sweden; cecilia.stenfors@psychology.su.se; 4 Aging Research Center, Department of Neurobiology, Care Science and Society, Karolinska Institute, 171 77 Stockholm, Sweden

**Keywords:** COVID-19, greenness, mental health, societal change, social isolation, psychological factors, resilience

## Abstract

International data suggest that exposure to nature is beneficial for mental health and well-being. The restrictions related to the COVID-19 pandemic have created a setting that allows us to investigate the importance of greenness exposure on mental health during a period of increased isolation and worry. Based on 2060 responses from an online survey in Stockholm County, Sweden, we investigated: (1) whether the COVID-19 pandemic changed peoples’ lifestyle and nature-related habits, and (2) if peoples’ mental health differed depending on their exposure to greenness. Neighborhood greenness levels were quantified by using the average normalized difference vegetation index (NDVI) within 50 m, 100 m, 300 m, and 500 m buffers surrounding the participant’s place of residence. We found that the number of individuals that reported that they visited natural areas “often” was significantly higher during the pandemic than before the pandemic. Higher levels of greenness surrounding one’s location of residence were in general associated with higher mental health/well-being and vitality scores, and less symptoms of depression, anxiety, and perceived and cognitive stress, after adjustments for demographic variables and walkability. In conclusion, the results from the present study provided support to the suggestion that contact with nature may be important for mental health in extreme circumstances.

## 1. Introduction

Countries worldwide have taken action to control population movement in response to the COVID-19 pandemic. The aim of the restrictions has been to reduce disease transmission by minimizing physical contact between people. These measures, however, have affected a much wider range of societal aspects than the disease spread, with consequences on the economy, social relations, health related behaviors as well as being disruptive for services and education [1,2,3]. People that are already struggling because of low income, social isolation, or poor health are also likely to be the most vulnerable to the adverse effects of the pandemic-related societal actions. Among those are elderly people, who have the highest risk of severe COVID-19 infection and at the same time, are at greatest risk of social isolation as they are less likely to use online communication [1]. People with underlying mental health conditions may be at risk of ending up increasingly isolated and, consequently, experience impaired mental health, which may result in the development of severe depression/anxiety-related symptoms [1,4,5]. Isolation may also increase the problems with substance abuse and potentially affect the number of cases of family violence [1]. In addition, for individuals on low income or on precarious contracts, the effects of the pandemic may become particularly severe as they already have poorer health and an increased likelihood to be in insecure work without financial reserves [1]. All this has raised concerns about the mental health consequences that this period of time may have on public health [2,4,6].

The COVID-19 pandemic-related restrictions in Swedish society have been relatively mild compared to most European countries [3]. Still, during the spring of 2020, the population of Sweden was recommended to, as much as possible, work from home, limit their social contacts, and avoid the use of public transport. In addition, no social mass gatherings were allowed to take place and the majority of the flights and some lines of public transport were temporarily stopped. The activity of high schools and universities mostly took place though digital platforms. For the elderly, they were strongly advised to self-isolate at home, and in care-homes, the contact with non-staff was very limited. Since loneliness and isolation have been problems in Swedish society before the COVID-19 pandemic, concerns have been raised about the potential mental health effects of the pandemic restrictions. According to Statistics Sweden, approximately four percent of the adult Swedish population was estimated to be socially isolated in 2016–2017, and the loneliest ones were the elderly [7]. Loneliness is a concern for both physical and mental health providers, as research is increasingly linking social isolation to declining physical, mental, and emotional well-being [8].

During the time of increased uncertainty, nature around our homes may play a key role in mitigating against adverse mental health outcomes [2,9]. A myriad of scientific studies have suggested that experiencing greenness and nature is associated with mental health benefits [10]. Greenness exposure has been linked to increased positive affects [11,12,13], happiness [14], increased engagement in social interactions [6,15,16], enhanced sense of meaningfulness [17], decreased mental distress, and improved manageability of life tasks [15,18,19,20]. In addition, experience with nature has been shown to be positively associated with various aspects of human cognitive function, memory and attention, and impulse control as well as children’s school performance and creativity [21,22,23,24,25,26,27,28,29].

When the range and contexts of people’s movements are curtailed, greenness exposure close-by homes, either through windows or in the immediate neighborhood including balconies and domestic gardens, may become increasingly important for psychological restoration and support mental well-being [2,9,30]. Several studies have indicated that seeing greenness though windows may promote health and well-being by providing micro-restorative episodes that promote healing [31,32,33,34], facilitate psychological restoration [35], increase recovery from stressful events [36], and improve affective and functional well-being [37]. Furthermore, being able to enjoy green window views has been seen to positively affect an individual’s cognitive capacity [38] increase life- [39] and job-satisfaction [40], and promote fascination and a sense of being away from everyday life [41]. In a recent study, Dzhambov et al. (2020) [9] reported that university students in Bulgaria, who were forced to self-isolate at home because of the COVID-19 restrictions, but had abundant greenery visible from their home windows or in the neighborhood, showed both reduced symptoms and rates of depression and anxiety. Spending time in a domestic garden has, among other health effects, been associated with reduced depression and anxiety [42,43,44]. However, it is important to remember that having a domestic garden is also strongly associated with socioeconomic status [45]. Still, the perceived restrictiveness of private gardens has been reported to rank higher than that of other private spaces [46], and gardening has been associated with reduced anxiety, depression, and many other mental health benefits [42,47].

Several studies have already reported an increased number of visits to urban natural areas such a parks and urban forests during the COVID-19 restrictions [48,49]. A recent Japanese study also found that increased exposure to neighborhood greenness and the frequency of green area use during the pandemic was positively associated with levels of self-esteem, life satisfaction, and subjective happiness as well as negatively associated with depression, anxiety, and loneliness [2]. In the present study, we investigated the possible changes in people’s habits of visiting nature during the spring of 2020, and also whether the mental health estimates during the same period of time were associated with the exposure to neighborhood greenness and to the nature-related behaviors in the adult population of Stockholm County, Sweden. The study was based on 2060 self-reported responses to a questionnaire sent out in June 2020.

## 2. Materials and Methods

### 2.1. Study Design and Sampling

Between 5 June and 1 August 2020, we conducted an online survey among adult residents (≥20 years) in three urban municipalities, eight suburban municipalities, and one rural municipality (see Supplementary Material Part A) in Stockholm County. The Swedish Population Register [50] provided the postal address data of the potential study participants (5000 men and 5000 women, randomly selected within the municipalities). We approached the potential participants through written requests to participate in a survey on greenness and mental health. The potential study group received a link to a web-questionnaire in Questback (https://www.questback.com, accessed on 15 May 2020). This survey was administered in Swedish and included questions about sociodemographic factors, daily activities and habits, mental health, and the neighborhood walkability (see the English translation of the questions and response alternatives that the present study was based on in Supplementary Material Part B). The participants were only able to submit their responses once. Two thousand and sixty (2060; about 20%) individuals submitted their survey responses. In general, the study design and procedure followed the general principles outlined in the Declaration of Helsinki. By filling in the survey, the study participants agreed that their personal information was processed and stored according to the General Data Protection Regulation of the European Union. The study was approved by the Swedish Ethical Review Authority.

### 2.2. Exposure Assessment

We transformed the participant addresses to geographic coordinates by using Open Streetmap (www.openstreetmap.org, accessed on 18 October 2020) and GIS (geographic information system) software Qgis (www.qgis.org, accessed on 18 October 2020). In about 10% of cases, when Open Streetmap was unable to find the address coordinates, the addresses were geocoded manually by using Google Maps in Chrome software.

#### Greenness Exposure

Normalized difference vegetation index (NDVI) [27], derived from Landsat 8 composite images (at a resolution of 30 × 30 m), was used to estimate residential greenness. NDVI is a remotely sensed measurement obtained by visible red (RED) and near infrared (NIR) radiation interacting with photosynthetic tissue in plants, and is calculated using the formula: NDVI = (NIR − RED)/(NIR + RED). NDVI values range from −1 to +1, where higher values indicate more green vegetation foliage. To avoid the possible effects of local level cloud contamination (resulting in false low values) and the year-specific variation (due to, for example, inter-year differences in precipitation), we used the highest NDVI value of the three consecutive years (2017, 2018, and 2019), within 50 m, 100 m, 300 m, and 500 m buffers surrounding the participants’ place of residence. To avoid underestimation of the NDVI exposure values, we excluded surfaces covered with water (represented by negative NDVI values) from the buffer areas.

### 2.3. Other Variables

#### 2.3.1. Walkability

We used the Health by Design questionnaire “How Walkable is Your Neighborhood?” (www.healthbydesignonline.org, accessed on 18 October 2020) to acquire the neighborhood walkability scores (Supplementary Material Part B). Since the walkability values in Swedish cities are generally very high, the thresholds recommended by the authors of the survey for categorization of the responses were not suitable. Instead the walkability scores were analyzed either as a continuous variable or categorized according to the interquartile level.

#### 2.3.2. Sociodemographic Variables

Based on available literature, we identified factors previously considered as potential confounders or effect modifiers in studies investigating associations between mental health outcomes and residential greenness. The data from the Swedish Population Register contained, in addition to the personal addresses, information about the age and sex of the potential participants. Information about the individuals’ education level, annual income, and ethnicity were collected in the survey (Supplementary Material Part B).

#### 2.3.3. Frequency of Nature Visits

We estimated the individual level frequency of nature visits by using the participants’ responses to the questions: “On average, how often did you visit nature areas such as parks/forests/bodies of water during the summer months before the COVID-19 pandemic?”; “On average, how often did you visit nature areas such as parks/forests/bodies of water during the winter months before the COVID-19 pandemic?”, and “During the COVID-19 pandemic, on average, how often did you visit nature areas such as parks/forests/bodies of water?” (see Supplementary Material Part B). The response alternatives to the above questions included: “Every day”, “One to several times per week”, “One to several times per month”, “One to several times per year”, and “Never”.

In Table 1 and Table 2, we present the proportion of study participants that visited natural areas “often”. This proportion was obtained by pooling (separately for each of the questions above) the proportion of individuals that responded with “Every day” and “One to several times per week” (all the other responses were pooled to the proportion of individuals that visited nature areas “seldom”). Since the proportion of people that visited natural areas “often” before the COVID-19 pandemic differed between summer (81%; generally acknowledged as April–September) and winter (54%; October–March), while the period of the COVID-19 pandemic included both “winter” and “summer” months, transformation of the data was necessary to make the before and during pandemic estimates comparable. We estimated that in June, when the majority of the study participants (about 95%) responded to the survey, the period of epidemic had consisted of approximately 1.5 winter (middle-February–March) and two summer months (April–June). Accordingly, we combined the respective responses to the questions about the frequency of nature visits during winter and summer before the COVID-19 pandemic into a time-weighted estimate (= (1.5/3.5 ∗ frequency of nature visits during summer) + (2/3.5 ∗ frequency of nature visits during winter)), and used this estimate as a proxy for how often the respondents would have been expected to visit nature during a corresponding time period if the pandemic had not occurred.

In cases when a participant had responded with “One to several times per year” or “Never” to a question about the frequency of nature visit before and respectively during the pandemic, this inquiry was followed by a question about the reasons for not visiting the nature areas more often (“What was your reason for, before/during the COVID-19 pandemic, not to visit nature areas more often?”). If the query about nature visit frequency was answered with “Every day”, “One to several times per week”, or “One to several times per month”, the query was followed by the inquiry of: “What was your reason to visit nature areas before/during the COVID-19 pandemic?”, and thereafter “What kind of natural areas did you visit during the COVID-19 pandemic and how often?” (see Supplementary Material Part B for the alternative responses).

#### 2.3.4. Alcohol Consumption

Data about alcohol consumption before and during the COVID-19 pandemic was estimated by combining the responses to: “How often did you drink alcohol before the COVID-19 pandemic/during the COVID-19 pandemic?” and to: “How many glasses/day did you typically drink when you drank alcohol before the COVID-19 pandemic/during the COVID-19 pandemic?” (one standard glass corresponds to 12 g of alcohol [51]; Supplementary Material Part B). Definitions of threshold levels for harmful alcohol consumption vary largely between the recommendations from different studies and authorities [51,52,53,54,55]. We set the amount of ≤7 standard glasses of alcohol/week for women and ≤10 standard glasses of alcohol/week for men as the threshold for “low risk for alcohol-related health problems” (used in, for example, [56]). Individuals with estimated consumption higher than this value were thus categorized as having “an increased risk for alcohol-related health problems”.

#### 2.3.5. Physical Inactivity

The “sitting score” was derived from the answers to the classic International Physical Activity Questionnaire—Short Form (IPAQ-SF). The sitting score was estimated from the responses to the questions: “Before the COVID-19 pandemic/During the COVID-19 pandemic, how much time did you usually spend sitting down on an average day during a regular week?” (see Supplementary Material Part B). The reason for using the sitting score rather than the total physical activity scores was that a comparably larger proportion of data was missing among the physical activity data than among the sitting scores, thus making it impossible to estimate the total physical activity scores for as many people as we had the physical inactivity data.

### 2.4. Outcome Variables

#### 2.4.1. RAND-36: Mental Health/Well-Being and Vitality Scores

The RAND-36 was originally developed to measure health related quality of life [57]. In the present study, we used the subscales of mental health and well-being (four items), and vitality (four items) (for more information about scoring and questions see Supplementary Material Part B). We computed the mean scores (according to [57] for each of the subscales and used these in the further analyses. Both subscales measured symptoms during the last month with higher values corresponding to beneficial mental health effects.

#### 2.4.2. SCL90: Core Depression and Anxiety Symptoms

We used the core depression subscale from the Hopkins Symptom Checklist 90 to estimate the occurrence of depressive symptoms (sum of the scores for six items; see Supplementary Material Part B). Due to space limitations in the questionnaire, the anxiety scores were only based on two items from the SCL anxiety subscale (SCL-90-CD; see Supplementary Material Part B) [58,59]. The excluded items of the original anxiety subscale focused on symptoms related to repetitive behaviors (such as repetitive washing, control behaviors), avoiding public places, and being uncomfortable eating and drinking out (e.g., in bars or restaurants). We judged these inquires less relevant for capturing anxiety symptoms during the COVID-19 pandemic, since such behaviors were part of the behavioral recommendations from the public health authorities in order to restrict COVID-19 transmission. Both outcome measures (depression and anxiety) measured symptoms during the last week. Lower subscale values correspond to beneficial mental health effects (i.e., a lack of the respective symptoms).

#### 2.4.3. Perceived Stress Scale-PSS

We estimated the perceived stress by using the sum of the scores of a six-item version of the Perceived Stress Scale [60]. The included questions focused on symptoms experienced during the last month and based on the classic PSS four-item short scale with two additional items, which have shown consistently high factor loadings in prior studies [61] (see Supplementary Material Part B for more information about scoring and included questions). Lower values correspond to beneficial mental health effects.

#### 2.4.4. The Cognitive Stress Score

We used the average values of the cognitive stress scale, originating from the Stress Profile [62], to estimate cognitive stress symptoms, experienced during the last month, in our study participants (see Supplementary Material Part B). The four questions target problems with concentration, memory, thinking clearly, and making decisions, and have been found to be associated with occupational stressors and objective executive cognitive functioning in prior work [63,64]. Lower values correspond to beneficial mental health effects.

### 2.5. Statistics

We conducted all statistical analyses by using the Stata 14.2 software (StataCorp LLC, College Station, TX, USA). The significance levels for differences between time periods (before resp. during the COVID-19 pandemic) and between groups were calculated by using Pearson chi-square tests for categorical variables and *t*-tests for continuous variables.

The associations between NDVI and the mental health outcomes were investigated by linear regression models, estimating beta coefficients (β) and 95% confidence intervals (95% CI). We used the stepwise forward regression modeling approach, based on change-in-estimates criteria, to identify covariates to include in the models. In the final model, covariates associated with the outcome at the 0.1 significance level were included [65]. These covariates were: age, sex, income, alcohol consumption (during the pandemic), physical inactivity (during pandemic), and the neighborhood walkability index. The estimate of education was not associated with any of the mental health outcomes (*p* > 0.5 in all cases) and was therefore excluded from the final model. Models were fitted for each mental health outcome separately. The linearity of the associations between NDVI exposure and mental health estimates was investigated through both visual observations of the graphically depicted mental health estimate and NDVI association, and by restricted cubic spline (RCS) linear regression models with three knots positioned according to Harrell’s recommended percentiles [66] on the NDVI scale in the 50 m buffer. The models with and without the RCS were compared using the log likelihood test for model fit. While the fully adjusted models in general showed a good fit for linear models (the curvilinear values did not appear significant), the visual examination of the original graphically depicted association between a mental health estimate and NDVI suggested a possible change in direction in the association somewhere in the range of 0.45 ≤ NDVI ≤ 0.50. Thus, we also decided to explore the associations by using two separate linear spline models with a knot at an NDVI value of 0.45 and 0.5, respectively. The significance level was set at *p* < 0.05 in all multivariate analyses estimating the association between NDVI and the respective outcomes.

## 3. Results

### 3.1. Descriptive Statistics, General

The general background statistics are shown in Table 1. The median NDVI exposures within 50 m, 100 m, 300 m, and 500 m buffers were 0.44, 0.45, 0.47, and 0.46, respectively. No differences regarding the NDVI exposure within 50 m buffers were detected in association with sex and age group. High income and education as well as high walkability tended to be more likely at low NDVI values. High alcohol consumption was more likely at low NDVI values. Being born in Sweden or in the rest of Scandinavia and the Baltic States increased the likelihood of living in a greener area compared to having any other country of birth. The frequency of people that visit natural areas “often” was higher at high neighborhood NDVI levels, and having a high sitting score, was less likely at high NDVI exposure.

### 3.2. Frequency of Nature Visits Pre-Pandemic versus during the Pandemic

On average, the percentage of people that reported that they visited natural areas “often” in our study population, was 13 percentage points higher during the COVID-19 pandemic than before the pandemic (t = −14.78, *p* < 0.0001; Table 2). Further analyses showed that this increase in the frequency of nature visits occurred in almost all population subgroups independently of age, sex, education, ethnic origin, neighborhood walkability, and the NDVI estimates (Table 2). However, this increase was six percentage points higher in the population younger than 70 years than in the population that were 70-years or older (confidence interval (CI) 95% 1.5, 10.4; *p* ≤ 0.009), 10.7 percentage points higher in individuals with higher education than in those with primary education (CI 95% 3.1, 18.4; *p* ≤ 0.006), and 5.8 percentage points higher in the population born in Sweden or in other Scandinavian/ Baltic countries compared to the population born elsewhere (CI 95% 0.2, 11.4; *p* ≤ 0.0411) (Supplementary Material Part C; Table S1.C for all between group differences).

There were no significant differences in the responses to the questions: “What was your reason, before the COVID-19 pandemic, not to visit nature areas more often?” and “What was your reason, during the COVID-19 pandemic, not to visit nature areas more often?” (Supplementary Material Part C; Table S2.C).

The proportion of individuals that responded with “often” or “very often” to the questions: “What was your reason to visit nature areas before the COVID-19 pandemic versus during the COVID-19 pandemic?” as well as the direction of the between period differences is reported in Table 3. The proportion of people that often visited natural areas for physical activity, to see other people, and because it was good for their health was significantly higher during the pandemic than before the pandemic. The proportion of the study population that reported that they often visited nature areas for reasons such as to recover from stress, to experience silence/nature sounds, to relax, to enjoy the beauty of nature, to be alone, to clear my head/think clearly, and because it is part of my regular transportation route, was significantly lower for the during-pandemic period than for the before the pandemic period. There were no significant between-period differences concerning the response alternatives: to be in the fresh air, for social reasons, for spiritual experiences, because somebody else told me to do that, and because my work requires it.

The proportion of individuals that responded with “often” or “very often” to an alternative of the question: “What kind of natural areas did you visit before and resp. during the COVID-19 pandemic?” are shown in Table 4. According to our data, the use of private gardens and nature reserves was significantly higher, and the use of the parks, freshwater bodies, beaches, and green play parks significantly lower during the pandemic than before the pandemic. The use of forests did not show any significant changes.

### 3.3. Alcohol Intake in the Study Population Pre-Pandemic resp. during the Pandemic

The average weekly consumption of alcohol was significantly higher during the pandemic than before the pandemic in the total sample as well as in several subgroups (see Table 5). The percentage of individuals with daily alcohol intakes above the low risk level were significantly higher during the pandemic than before the pandemic in almost all the subgroups (Table 5).

### 3.4. Sitting Score

The average weekly sitting scores were reported to be significantly higher during the COVID-19 pandemic than before the pandemic in all subgroups (Table 6). We did not find any significant between-group differences in the percentage points of change.

### 3.5. Associations between Normalized Difference Vegetation Index (NDVI) and the Mental Health Estimates

The mental health estimates did not differ between the individuals that were exposed to NDVI levels (within 50 m buffers) below versus above the median (Table 7). However, when the NDVI exposure was stratified by walkability measures (either below or above the median levels), the mental health estimates significantly differed between the high and low NDVI exposure groups, but only when the walkability was low (Supplementary Material Part C; Table S3.C).

**Figure 1:**
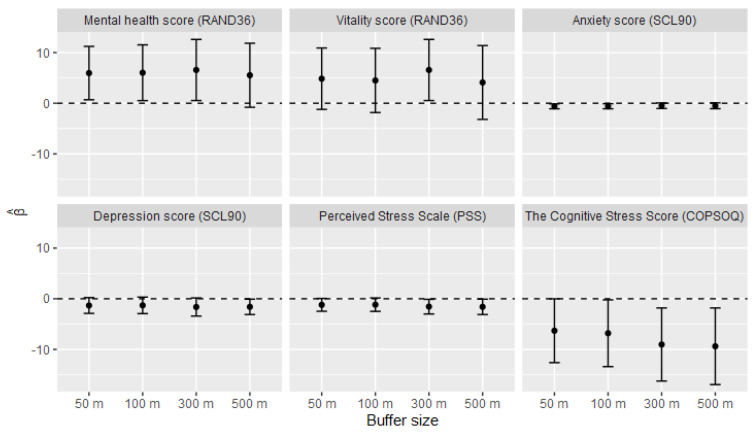
Mental health estimates associated with NDVI within different buffer sizes (models adjusted as in Table 6) (95% CI).

No mental health estimates were associated with NDVI in the non-adjusted models or in models only adjusted for sex and age (Supplementary Material Part C; Table S4.C). The results of the fully adjusted models are shown in Table 8 (for 50 m buffers including the estimates for all covariates) and in Figure 1 (corresponding numerical values are shown in the Table S5.C in Supplementary Material Part C). In fully adjusted models, increased NDVI was associated with better mental health regardless of the estimate; however, the effect and significance levels differed between different buffer sizes. While the mental health and anxiety scores showed significant associations with the NDVI values at smaller buffer sizes (i.e., 50 m and 100 m), the association with depression and vitality scores as well as with the perceived stress scale were significant only at larger buffer sizes (300 m and/or 500 m). The association between the cognitive stress scale and NDVI was significant at all buffer sizes. Income and walkability appeared to be the covariates with the largest confounding effect in all cases and were positively associated with the mental health estimates. Being male, older, born in Sweden as well as having low physical inactivity score, consuming alcohol below the “low risk” level and visiting natural areas “often” were generally associated with better mental health.

**Figure 2:**
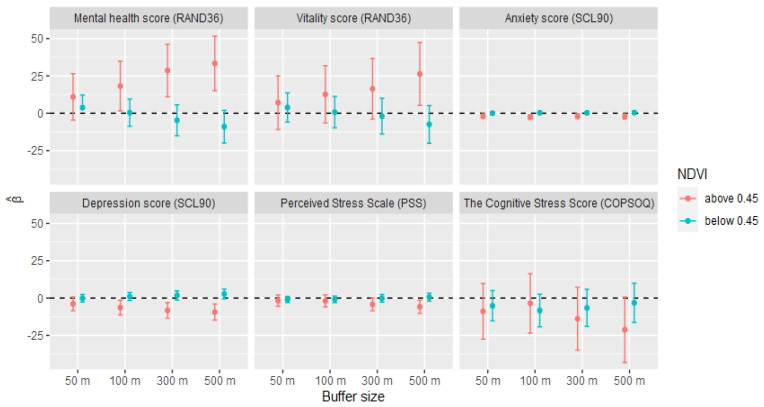
Mental health estimates associated with NDVI within different buffer sizes (models adjusted as in Table 6). Fully adjusted linear spline models with the knot set to NDVI = 0.45 (95% CI).

The linear spline models with the knot at a NDVI value of either 0.45 (Figure 2) or 0.5 (Supplementary Material Part C; Table S6.C) revealed a pattern according to which only the NDVI values above the knot value were significantly associated with the mental health estimates.

## 4. Discussion

The number of individuals that reported that they visited natural areas “often” was significantly higher during the pandemic than before the pandemic in all population subgroups. However, this increase was significantly higher among those who were younger than 70 years (compared to those being older than 70 years), had university education (compared to the primary education), and were born in Sweden or in other Scandinavian/Baltic countries (compared to those born elsewhere). Visits to nature reserves and private gardens increased significantly during the pandemic, while visits to parks, water-related areas, and green play-parks decreased. The number of individuals with alcohol consumption above the “low-risk level” increased significantly during the pandemic compared to prior to the pandemic. High levels of greenness surrounding one’s location of residence were associated with better mental health in almost all mental health estimates analyzed in this study, after adjustments for demographic variables and walkability. Being female, physically inactive, born outside Sweden/rest of the Nordic countries, and consuming alcohol above the low-risk level was generally associated with lower mental health estimates. Higher age, walkability index, annual income, and visiting natural areas “often” was associated with better mental health.

Our study is largely in agreement with the findings from previous studies. For example, two recent publications from Japan and Bulgaria have reported that increased frequency of green area use and/or increased access to green views through the windows at home during the COVID-19 pandemic-related restrictions were associated with positive mental health effects [2,9]. In the present study, we did not investigate the effect of the green window views; however, we believe that using (among others) the rather small buffer sizes (such as 50 m surrounding the place of residence) did reflect the relative difference in the quantity of greenness that people were able to see through their windows. Generally, while the well-being-mental health (RAND36), vitality (RAND36), and anxiety (SCL90) scores in our study showed significant associations with the surrounding greenness when the buffer sizes were small (50 m and 100 m), the relationships between greenness and depression (SCL90) as well as between greenness and perceived stress scale (PSS) scores were significant only at larger buffer sizes (300 m and 500 m). This may indicate a difference in the mechanistic pathways regarding the relationship between various mental health estimates and greenness. It is possible that while in some cases the positive health effect may be a result of passively viewing nature, in other cases, it could be related to physical activity and movement in the surrounding areas. The cognitive stress scores showed a significant relationship with the surrounding greenness at all buffer sizes. This corresponds to previous findings on the positive impact of nature interactions as well as neighborhood greenness on cognitive functioning [23,29].

A multinational survey including responses from 77 different countries found that while the pandemic-related lockdowns significantly affected mental health, the contact with nature helped people to cope with these impacts [30]. Furthermore, the positive effect of nature was especially obvious in countries with strict lockdown restrictions. The nature of the mental health estimation tools used in our study does not allow for any retroactive analyses of mental health, thus we were not able to analyze the change in the mental health between the periods before the pandemic and during the pandemic. However, the effect of greenness on the mental health estimates showed a constant positive trend in the fully adjusted models, and this relationship was further strengthened by the self-reported high frequency of the nature visits.

We did not find any associations between the mental health outcomes and neighborhood greenness levels in our unadjusted models. This result was not surprising for several reasons. Socioeconomic status is known to be positively related to mental health [67,68,69], while the levels of neighborhood greenness may also differ depending on the socioeconomic factors [70,71]. In a previous study, we found that in Stockholm County, the direction of the association between area level greenness and socio-economy showed opposite trends, depending on the type of municipality in focus [72]. Generally, the central urban areas with high socioeconomic status had relatively low levels of greenness (compared to the urban areas with low socio-economy); while in the suburbs, areas with low socio-economic status had lower greenness levels (compared to suburban areas with high socio-economy). The municipalities that were included in the present study did differ in their degree of urbanization, which is (based on the opposite directional associations described above) likely to counteract the appearance of any obvious mental health–greenness trends in the unadjusted models. The walkability indexes, based on self-reported responses, strongly reflected the degree of area urbanization (Supplementary Material Part A). In groups exposed to higher neighborhood walkability as well as higher income, the positive association between neighborhood greenness and mental health was profoundly stronger.

Our investigative approach to use linear spline models with a knot at a NDVI value of 0.45 (based on visual observations of the relationships between mental health scores and NDVI values) revealed a pattern according to which only the NDVI values above the knot value were significantly associated with the mental health estimates. One possible interpretation of this is that the quantity of greenness only affects mental health above a specific (and in this case, rather high) threshold value. However, as the NDVI values are lowest in central Stockholm, where the socioeconomic status of the residents is rather high (and higher socioeconomic status shows a positive relationship with the mental health estimates), this could simply be an effect of the location-specific differences in the interactions between the levels of greenness, socio-economy, and the mental health scores. Since no other studies have previously used this approach, we are unable to compare our results to the data from others.

The participants of the present study reported that they visited natural areas significantly more often during the spring of the pandemic than they would have been expected to do if the pandemic had not happened. The use of the private gardens (including privately owned summerhouses) was reported to have significantly increased during the pandemic compared to before the pandemic period. This is not surprising, as using gardens is among the easiest and safest ways to get away from the indoor environment without the need of encountering the general public. Private gardens may also have provided opportunities for physical activity (for example, in form of gardening work) for people that avoided visiting gyms or other sporting facilities because of the COVID-19 pandemic (many of these facilities in Sweden were open for public use during the spring of 2020, however, the degree of use decreased markedly, especially among the elderly).

A recent study from Oslo, Norway reported intensified activity of both pedestrians and cyclist on trails with higher greenness during the COVID-restrictions during the spring of 2020 [49]. Furthermore, the trail use was affected by the trail accessibility and social distancing preferences, as the trail remoteness significantly increased the use of a trail [49]. These results partly reflect our findings. The participants in the present study reported that they visited nature reserves more frequently during the pandemic than before the pandemic. Since nature reserves tend to generally be found in more remote locations than other dedicated urban green areas, it may reflect the tendency of people to find places where the risk of encountering others is low. In contrast to the studies from Norway [49] and Germany [48], where the authors also found increased pedestrian activity in the city parks and peri-urban forests, the participants of the present study reported that they visited parks less often during the pandemic than before the pandemic, while the frequency of forest visits did not change. Since these studies used different methods to register the indications of the movements of the people (mobile tracking [49], interviews [48], and here, self-reported questioners), the data are not comparable as such, and is in our case, strongly dependent on the characteristics of the population group that chose to respond to the questionnaire. However, several of the participants in the present study had commented in the free text that they did not feel secure when outside because of the pandemic, which could partly explain why people chose to visit areas farther away from crowds. Examples of such free text responses were: “Other people are coming too close, I must choose more isolated walkways”, “I am in the risk group”, “I do not want to be close to people”, “The areas I have within walking distance are overcrowded and people do not keep distance”, “Lots of people in many natural areas”, etc.

The reasons that were reported for not visiting natural areas did not differ between the pre- and during-pandemic period. However, significantly more people reported that they visited natural areas for physical activity, to see other people, and because it was good for their health during the pandemic than before the pandemic. Significantly fewer individuals reported that they visited natural areas to recover from stress, to experience silence/nature sounds, to relax, to enjoy the beauty of nature, to be alone, to clear their head/think clearly, and because it is part of their regular transportation route during the pandemic than before the pandemic. It is likely that rather than the population of Stockholm County losing their ability to enjoy the sounds and beauty of nature, these responses reflect a change of focus during the pandemic. Being able to experience the existence of other individuals as well as having an opportunity for physical activity may have become more important during this time-period, characterized by decreased social interactions, increased time spent sitting, and restricted access to sporting facilities/activities than the aesthetics of nature. Furthermore, since many chose to work from home, the number of people that visited natural areas because it was part of their regular transportation route was expected to decrease. Some of the reasons for visiting natural areas during the COVID-19 pandemic given in the free text stated that natural environments helped people to: “activate their children”, “be more creative”, “be able to meet friends while avoiding indoor environments”, “be able to meet relatives”, “reduce the risk for obesity”, “have a sense of freedom”, “keep their body active”, “get to know their immediate environment better, now when they spend more time at home”, “see something else than their flat” as well as “because there is nothing else to do”.

There has been a growing concern in the literature that the pandemic-related social isolation may lead to an increase in alcohol consumption and/or abuse [73,74,75,76]. Several studies have pointed out that the stress associated with increased loneliness and potential economic difficulties or domestic relationship problems may serve as significant triggers for alcohol use and may lead to an increase in the prevalence of alcohol use disorder and alcohol-related harms [74,76,77,78]. In our present study, we found indications of increased alcohol consumption during the pandemic. In particular, individuals of the male sex and younger than 70-years seemed to have significantly increased their weekly alcohol intake. It might therefore be important to acknowledge the potential impact of alcohol-related problems associated with the COVID-19 pandemic, and discuss the necessary strategies, which would help society cope with these problems. These could include precautionary measures, like providing support for coping with stress and social isolation. However, it might also be necessary for health care institutions to prepare for an increase in the demand for their services due to alcohol-related problems [74].

Urbanization has decreased human contact with nature, but increased the potential of the spread of pandemics [79,80]. Previous studies have reported the health benefits of visiting natural areas [81] and the capacity of nature to buffer the negative health effects in people suffering from social isolation [82] or other stressful life events [19]. Thus, we agree with the conclusion of Soga et al. [2] and Pouso et al. [30] that urban nature can be used as a nature-based solution for improved public health. Our study, together with the results from others, may help decision makers to increase their preparedness for possible long-term effects of the COVID-19 pandemic as well as to mitigate the negative effects of future lockdown strategies by ensuring that people have access to nearby greenery and in this way, increasing their resilience when suffering by stress or social isolation.

An important question in the context is how or if the increased interest in urban nature will affect the population and society after the pandemic has passed. In the present paper, we saw that the number of people that visited nature areas “often” increased significantly from the pre-pandemic period to the during-pandemic period, implying that a considerable proportion of the population had changed their nature visiting routines. If these individuals perceived that their increased time in the nature also increased their sense of well-being, they may be motivated to retain their new routines when the pandemic has passed. The reasons for not visiting natural areas did not differ significantly when the responses from before the pandemic were compared to the responses from during the pandemic (see Supplementary Material Part C, Table S2.C). This may indicate that the group of people with no previous nature visiting routines did not change their priorities during the COVID-19 pandemic-related restrictions, and may thus also be less likely to be influenced by any future trends and recommendations. However, different post-pandemic policies in the society may affect the behavior of the population. For example, if the employers will, after the pandemic, allow working from home in a larger extent than before the pandemic, people may have more time for nature walks during their workdays, since they do not need to spend time on commuting. The media interest regarding the importance of visiting nature areas has been high during the COVID-19 pandemic (i.e., [83,84,85]), which may have influenced how the society (both the general population and the policy-makers) values urban nature, and thus, may also affect our future urban planning priorities.

Some limitations should be considered when interpreting the results from our study. All presented data, except for the objectively estimated NDVI values, was self-reported. Since the data sampling was cross-sectional, we chose to make inquiries about certain behaviors for both the pre-pandemic and during-pandemic periods. This design gave us an opportunity to discover the potential changes in the behaviors; however, we are aware that recall-bias may have affected the registered responses. Due to the limited time to conduct the study, we decided to only send out the recruiting letters once, but approach a rather large number of people (10,000 individuals). According to our previous experience, a response frequency of about 15% was expected. Despite that, the actual response frequency (about 20%) slightly exceeded our expectation; the large majority of our sample (randomly chosen within pre-decided municipalities) did not respond. This was likely to have influenced the composition of the sample with a bias toward responses from people interested in nature and with overrepresentation of highly educated individuals and people with good socio-economy.

## 5. Conclusions

Our study provided evidence that contact with nature may be important for mental health in extreme circumstances. The COVID-19 related societal restrictions affected the behavior of the population of Stockholm County by increasing the frequency of nature visits, but also led to increased alcohol consumption and more hours spent sitting still. Neighborhood-level greenness seems to affect several aspects of psychological well-being, especially in combination with good walkability.

## Figures and Tables

**Table 1:** Background characteristics related to normalized difference vegetation index (NDVI) values in 50 m buffers below and resp. above the median levels (NDVI = 0.46).

Variable Name	NDVI Low	NDVI High	*p*-Value
	N (%)	N (%)	
Sex			0.232
Females	583 (51)	556 (49)	
Males	447 (49)	474 (51)	
Age group			0.865
<70 years	842 (50)	839 (50)	
≥70 years	188 (49)	192 (51)	
Educational level			0.000
Primary education	42 (37)	71 (63)	
Secondary education	247 (45)	307 (55)	
Higher education	734 (53)	644 (47)	
Income			0.000
<225 tSEK	111 (40)	169 (60)	
225–450 tSEK	304 (45)	377 (55)	
>450 tSEK	600 (56)	467 (44)	
Country of Birth			0.019
SSB *	897 (49)	930 (51)	
All other countries	127 (57)	94 (43)	
Alcohol consumption			0.019
Below risk	831 (48)	885 (52)	
Above risk	183 (57)	136 (43)	
Walkability			0.000
Low	243 (38)	397 (62)	
Average	323 (48)	349 (52)	
High	454 (63)	270 (37)	
Sitting score			0.017
Low	324 (47)	371 (53)	
Average	338 (49)	356 (51)	
High	333 (54)	280 (46)	
Frequency of nature visits			0.020
Often	792 (49)	835 (51)	
Seldom	238 (55)	195 (45)	

* SSB—Sweden, rest of Scandinavia and the Baltic States.

**Table 2:** The percentage of people that reported that they visit natural areas “often” (* *p* < 0.05; CI—confidence interval).

Population			Before COVID-19	During COVID-19	Difference
		n	% (95% CI)	% (95% CI)	% (95% CI)
Total		2059	65.98 (64.27; 67.69)	79.02 (77.26; 80.78)	+13.04 (14.77; 11.31) *
Age	<70 years	1680	63.43 (61.52; 65.34)	77.56 (75.56; 79.56)	+14.13 (16.08; 12.18) *
	≥70 years	379	77.31 (73.71; 80.9)	85.49 (81.93; 89.05)	+8.18 (11.83; 4.53) *
Sex	men	920	62.25 (59.58; 64.92)	73.91 (71.07; 76.76)	+11.66 (14.21; 9.11) *
	women	1139	69 (66.8; 71.19)	83.14 (80.97; 85.32)	+14.15 (16.5; 11.79) *
Education	primary	113	67.89 (60.06; 75.71)	71.68 (63.25; 80.12)	+3.79 (10.66; −3.08)
	secondary	554	64.7 (61.32; 68.07)	75.63 (72.05; 79.22)	+10.93 (14.3; 7.56) *
	higher	1378	66.54 (64.48; 68.59)	81.06 (78.99; 83.13)	+14.52 (16.64; 12.41) *
Ethnic origin	born in SSB ^1^	1806	67.32 (65.51; 69.14)	80.95 (79.14; 82.77)	+13.63 (15.42; 11.84) *
	not born in SSB ^1^	221	55.87 (50.82; 60.91)	64.23 (58.2; 70.26)	+8.36 (14.39; 2.34) *
	both or one of the parents from SSB ^1^	1806	67.32 (65.51; 69.14)	80.95 (79.14; 82.77)	+13.63 (15.42; 11.84) *
	neither mother nor father from SSB ^1^	246	55.87 (50.82; 60.91)	64.23 (58.2; 70.26)	+8.36 (14.39; 2.34) *
Residential area walkability	Poor (lowest tertial)	640	64.87 (61.8; 67.93)	80.31 (77.22; 83.4)	+15.45 (18.64; 12.26) *
	Good (2nd tertial)	672	65.99 (63; 68.97)	78.87 (75.77; 81.96)	+12.88 (15.91; 9.85) *
	Very Good (highest tertial)	724	66.63 (63.72; 69.54)	77.9 (74.87; 80.93)	+11.27 (14.12; 8.41) *
Residential NDVI within a 50 m buffer	Low NDVI (0.236–0.343)	687	64.82 (61.83; 67.8)	76.42 (73.24; 79.6)	+11.6 (14.74; 8.46) *
	Average NDVI (0.344–0.431)	686	65.54 (62.51; 68.56)	79.3 (76.26; 82.34)	+13.77 (16.75; 10.78) *
	High NDVI (0.5–0.559)	686	67.6 (64.71; 70.48)	81.34 (78.42; 84.26)	+13.74 (16.61; 10.88) *

^1^ SSB—Sweden, rest of the Scandinavia and the Baltic States.

**Table 3:** The proportion of individuals that responded with “often” or “very often” to the questions: “What was your reason to visit nature areas before resp. during the COVID-19 pandemic?”.

	Before COVID-19	During COVID-19	Difference between before and during COVID-19	
n = 1928	%	%	*p*<	Direction
To be in the fresh air	80.0	80.3	0.716	-
To recover from stress	36.0	33.8	0.028	↓
For physical activity	69.7	72.1	0.028	↑
To experience silence/nature sounds	45.9	38.8	0.001	↓
For social reasons	22.0	21.3	0.456	-
To see other people	11.9	14.6	0.001	↑
To walk my dog (or other pet)	12.8	12.8	0.317	-
To relax	62.4	52.5	0.001	↓
To enjoy the beauty of nature	64.8	58.7	0.001	↓
To be alone	18.6	15.7	0.001	↓
For spiritual experiences	8.5	8.7	0.651	-
Because somebody else told me to do that	3.2	3.6	0.225	-
Because it is good for my health	68.8	72.5	0.001	↑
To clear my head/think clearly	37.1	35.0	0.016	↓
Because my work requires it	3.1	3.4	0.331	-
Because it’s part of my regular transportation route	10.5	8.0	0.001	↓

**Table 4:** The proportion of individuals responding with “often” or “very often” to an alternative of the question: “What kind of natural areas did you visit before and resp. during the COVID-19 pandemic?”.

	Before COVID-19	During COVID-19	Difference between before and during COVID-19	
n = 1928	%	%	*p*<	Direction
Private garden	40.0	41.3	0.046	↑
Park	43.7	39.8	0.016	↓
Forest	51.5	52.6	0.274	-
Freshwater bodies of water	41.8	39.6	0.020	↓
Saltwater beach/boating	16.8	15.5	0.020	↓
Nature reserve	27.0	29.3	0.003	↑
Green play parks	15.6	14.2	0.039	↓

**Table 5:** Alcohol intake in the study population during the pre-pandemic and resp. pandemic period.

		Mean Weekly Consumption (In Glasses)		Difference between before and during COVID-19		Individuals above the Low Risk Level (%) *		Difference between before and during COVID-19	
	n	Before COVID-19	During COVID-19	*p*	Direction	Before COVID-19	During COVID-19	*p*	Direction
Total	2025	3.83	3.95	0.022	↑	13.80	15.70	0.000	↑
Sex									
Men	903	4.48	4.66	0.036	↑	7.60	9.90	0.001	↑
Women	1122	3.32	3.38	0.276	-	18.80	20.40	0.034	↑
Age group									
<70 years	1658	3.67	3.79	0.005	↑	13.30	15.10	0.002	↑
≥70 years	367	4.55	4.71	0.180	-	16.30	18.40	0.032	↑
Education									
primary	110	3.81	2.64	0.436	-	14.40	13.50	0.320	-
secondary	546	3.97	4.03	0.554	-	12.30	15.60	0.001	↑
higher	1122	3.76	3.93	0.008	↑	14.30	15.90	0.014	↑

* The threshold for individuals above the “low risk” level is >10 glasses/week for men and >7 glasses/week for women.

**Table 6:** The average weekly sitting scores.

Subgroup	n	Mean Sitting Score		Between Period Difference		
		Before COVID-19	During COVID-19	Difference	*p*<	Direction
Sex						
men	889	6.7	7.3	0.6	0.001	↑
women	1111	6.6	7.1	0.5	0.001	↑
Age group						
<70 years	1642	6.9	7.5	0.5	0.001	↑
≥70 years	345	5.4	5.9	0.5	0.001	↑
Country of birth						
SSB	1780	6.7	7.2	0.5	0.001	↑
outside SSB	214	6.7	7.4	0.7	0.001	↑
Education						
primary	105	5.1	5.8	0.7	0.006	↑
secondary	537	6.4	7.0	0.6	0.001	↑
higher	1350	6.9	7.4	0.5	0.001	↑
Neighborhood walkability (tertials)						
poor	628	6.6	7.2	0.6	0.001	↑
good	656	6.6	7.2	0.5	0.001	↑
very good	704	6.7	7.2	0.5	0.001	↑
NDVI within 50 m						
low (0–0.29)	373	6.6	7.2	0.5	0.001	↑
average (0.3–0.49)	901	6.8	7.3	0.5	0.001	↑
high (0.5–1)	726	6.5	7.0	0.5	0.001	↑

**Table 7:** Average values of the mental health estimates stratified by NDVI exposure levels below and resp. above the median level within 50 m buffers (CI—confidence interval).

Mental Health Estimate	Average Score (CI 95%) at below Median NDVI	Average Score (CI 95%) at above Median NDVI	*p*<	Cronbach’s Alpha *
Mental health score (RAND36)	71.82 (70.735; 72.905)	73.007 (71.949; 74.065)	0.124	0.833
Vitality score (RAND36)	60.093 (58.839; 61.347)	61.116 (59.864; 62.368)	0.257	0.833
Anxiety score (SCL90)	1.231 (1.129; 1.333)	1.115 (1.023; 1.206)	0.096	N/A **
Depression score (SCL90)	5.906 (5.58; 6.232)	5.68 (5.377; 5.984)	0.320	0.900
Perceived Stress Scale (PSS)	8.732 (8.469; 8.996)	8.477 (8.229; 8.724)	0.166	0.755
The Cognitive Stress Score (COPSOQ)	31.55 (30.279; 32.821)	30.626 (29.352; 31.901)	0.314	0.888

* Cronbach’s alpha for the outcome scale; ** Cronbach’s alpha not available as the estimate consisted of two questions only.

**Table 8:** Association between NDVI within 50 m and the mental health estimates—fully adjusted model (* *p* < 0.05; CI—confidence interval).

Variable Name	Mental Health Score (RAND36)	Vitality (RAND36)	Anxiety Score (SCL90)	Depression Score (SCL90)	Perceived Stress Scale (PSS)	The Cognitive Stress Score (COPSOQ)
	β (95% CI)					
NDVI 50 m	5.951 (0.688; 11.213) *	4.842 (−1.219; 10.902)	−0.615 (−1.095; −0.136) *	−1.331 (−2.889; 0.227)	−1.202 (−2.465; 0.061)	−6.307 (−12.604; −0.01) *
Walkability	0.199 (0.136; 0.261) *	0.222 (0.15; 0.294) *	−0.016 (−0.021; −0.01) *	−0.056 (−0.074; −0.037) *	−0.042 (−0.057; −0.027) *	−0.149 (−0.224; −0.074) *
Sex						
male	ref.	ref.	ref.	ref.	ref.	ref.
female	−2.419 (−3.92; −0.919) *	−2.44 (−4.168; −0.711) *	0.146 (0.009; 0.283) *	0.461 (0.016; 0.905) *	0.586 (0.225; 0.946) *	3.033 (1.236; 4.83) *
Age	0.253 (0.203; 0.303) *	0.303 (0.246; 0.361) *	−0.024 (−0.029; −0.019) *	−0.066 (−0.081; −0.051) *	−0.069 (−0.081; −0.057) *	−0.309 (−0.369; −0.249) *
Annual Income	1.548 (1.1; 1.995) *	1.448 (0.933; 1.964) *	−0.156 (−0.197; −0.115) *	−0.55 (−0.682; −0.417) *	−0.249 (−0.357; −0.141) *	−1.735 (−2.271; −1.199) *
Physical inactivity	−0.554 (−0.791; −0.317) *	−0.952 (−1.225; −0.68) *	0.04 (0.018; 0.061) *	0.204 (0.133; 0.274) *	0.065 (0.008; 0.122) *	0.577 (0.294; 0.86) *
Frequency of nature visits						
seldom	ref.	ref.	ref.	ref.	ref.	ref.
often	3.638 (1.811; 5.466) *	6.685 (4.581; 8.79) *	−0.157 (−0.323; 0.01)	−1.004 (−1.545; −0.464) *	−0.617 (−1.055; −0.178) *	−2.6 (−4.784; −0.417) *
Place of birth						
Sweden	ref.	ref.	ref.	ref.	ref.	ref.
other SBC	1.577 (−1.975; 5.129)	2.351 (−1.739; 6.442)	0.03 (−0.294; 0.354)	−0.714 (−1.766; 0.338)	0.007 (−0.852; 0.865)	−2.854 (−7.109; 1.4)
rest of the Europe	−4.415 (−7.861; −0.97) *	−3.159 (−7.126; 0.809)	0.457 (0.144; 0.769) *	0.465 (−0.55; 1.481)	1.578 (0.746; 2.41) *	2.726 (−1.379; 6.831)
rest of the world	−2.963 (−6.159; 0.233)	−2.907 (−6.587; 0.774)	0.397 (0.105; 0.688) *	0.515 (−0.432; 1.461)	1.148 (0.383; 1.912) *	0.731 (−3.097; 4.558)
Alcohol consumption						
below the risk level	ref.	ref.	ref.	ref.	ref.	ref.
above the risk level	−3.09 (−5.105; −1.076) *	−3.449 (−5.769; −1.129) *	0.155 (−0.029; 0.338)	1.018 (0.421; 1.615) *	0.663 (0.178; 1.148) *	1.459 (−0.957; 3.874)

## Data Availability

The data presented in this study are available on request from the corresponding author. The data are not publicly available because it contains individual-specific health and sociodemographic data.

## Supplementary Materials

The following are available online at https://www.mdpi.com/1660-4601/18/6/3303/s1, Part A: Background characteristics of the municipalities. Table S1.A: Background characteristics of the municipalities involved in the study, Figure S1.A: The municipalities of Stockholm County. Part B: The selection of questions in the survey, relevant for the present study. Part C: Supplementary results. Table S1.C: Between group differences in the percentage of people visiting natural areas "often" pre-pandemic versus during the pandemic, Table S2.C: What was your reason, before/during the Covid-19 pandemic, not to visit nature areas more often?, Table S3.C: NDVI within 50m buffers and the average Mental Health scores, stratified by walkability, Table S4.C: Linear regression models adjusted for sex and age only, Table S5.C: Mental health estimates associated with NDVI within different buffer sizes (models adjusted as in Table 6), Table S6.C: Fully adjusted linear spline models with the knot set to NDVI = 0.5.
