# Supplementary material for: Mental Health, Greenness, and Nature Related Behaviors in the Adult Population of Stockholm County during COVID-19-Related Restrictions
Source: Int J Environ Res Public Health. 2021 Mar 23;18(6):3303. doi: 10.3390/ijerph18063303 (PMC8004976; doi:10.3390/ijerph18063303)
Supplement: Supplementary file 1 [file ijerph-18-03303-s001.pdf]

Part A - Background characteristics of the municipalities

Table S1.A. Background characteristics of the municipalities involved in the study

**Municipality**	**Number of participants**	**Average NDVI 50m (min; max)**	**Average walkability index (min; max)**
Danderyd	40	0.55 (0.35; 0.66)	81.27 (60.42; 100)
Huddinge	96	0.48 (0.15; 0.72)	78.31 (52.08; 96.88)
Järfälla	81	0.45 (0.16; 0.61)	81.24 (39.93; 100)
Lidingö	47	0.45 (0.15; 0.62)	83.95 (63.54; 96.88)
Sollentuna	91	0.46 (0.21; 0.64)	78.95 (50.00; 100)
Solna	114	0.43 (0.13; 0.63)	86.44 (48.96; 100)
Stockholm	1,254	0.38 (0.08; 0.77)	84.58 (35.42; 100)
Sundbyberg	74	0.35 (0.15; 0.64)	84.66 (62.50; 100)
Södertälje	61	0.49 (0.15; 0.73)	75.40 (48.96; 100)
Tyresö	60	0.51 (0.17; 0.68)	77.39 (43.75; 100)
Täby	98	0.46 (0.21; 0.67)	83.34 (48.96; 100)
Upplands Väsby	44	0.44 (0.21; 0.71)	80.72 (61.46; 100)

Supplementary material

Figure S1.A. The municipalities of Stockholm County

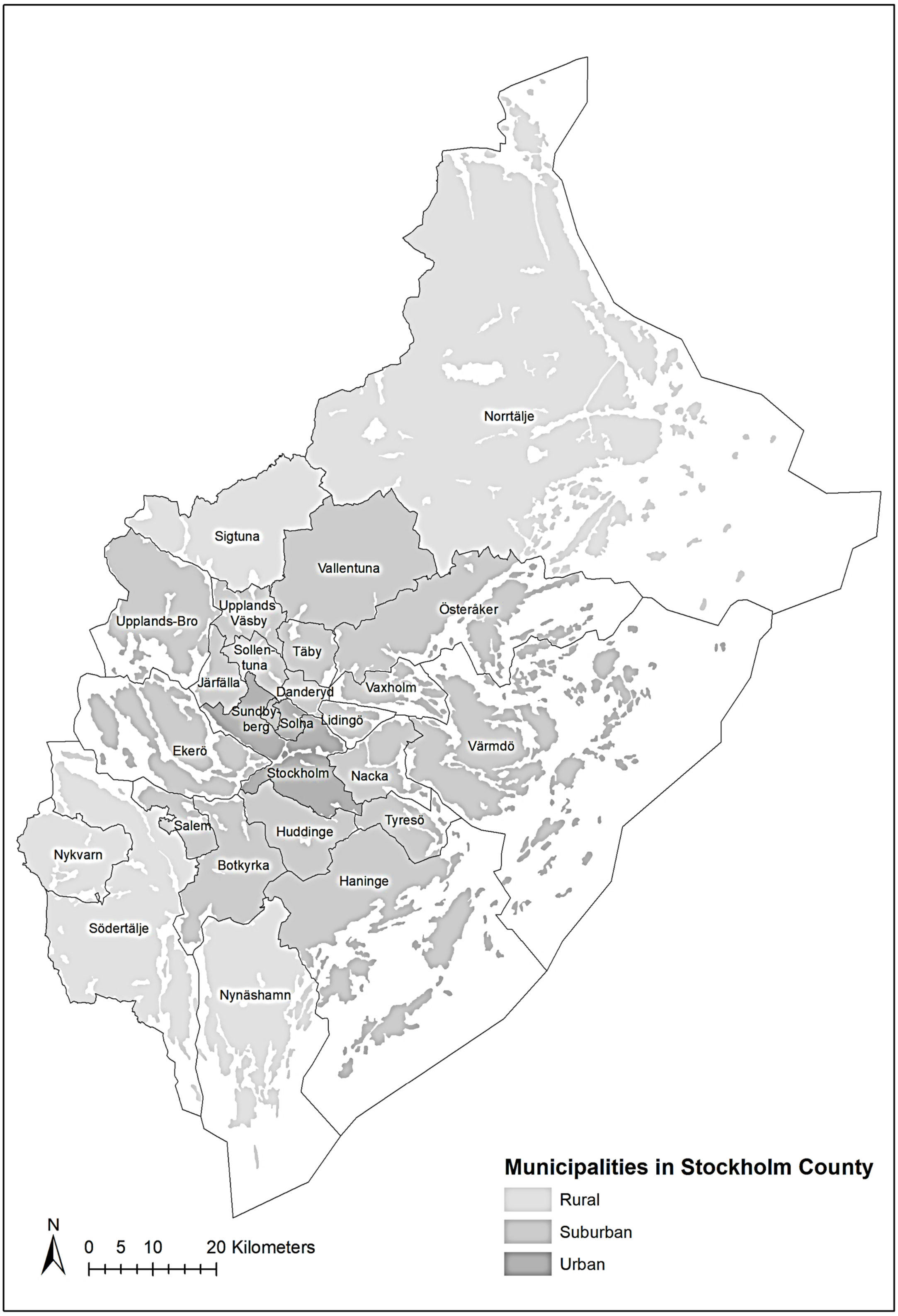

Supplementary material

Part B – the selection of questions in the survey, relevant for the present study

1. Background questions

39. What is your highest level of education?

Response alternatives:

1	2	3
primary	secondary	higher

40a. In which part of the world were you born?
40b. In which part of the world was your mother born?
40c. In which part of the world was your father born?

Response alternatives:

1	2	3	4
Sweden	Nordic/Baltic	Rest of the Europe	Rest of the world

41. What is your annual earned income before tax?

Response alternatives:

1	2	3	4	5	6	7
0 – 74 999	75 000 – 149 999	150 000 – 224 999	225 000 – 299 999	300 000 – 374 999	375 000 – 449 99	450 000 –

2. Nature visits

2.1 Frequency

(The number before the question reflects the question number in the survey)

15. On average, how often did you visit nature areas, such as parks/forests/bodies of water, during the summer months before the Covid-19 pandemic?

16. On average, how often did you visit nature areas, such as parks/forests/bodies of water, during the winter months before the Covid-19 pandemic?

20. During the Covid-19 pandemic, on average, how often did you visit a nature area?

Response alternatives:

1	2	3	4	5
Every day	One to several times per week	One to several times per month	One to several times per year	Never

2.2 Reason for not visiting the nature areas

17. What was your reason for, before the Covid-19 pandemic, **not** to visit nature areas more often? You can choose more than one answer. (Only those who responded with alternative 4 or 5 to question 15.)

21. What was your reason for, before the Covid-19 pandemic, **not** to visit nature areas more often? You can choose more than one answer. (Only those who responded with alternative 4 or 5 to question 20)

Response alternatives:

		0	1
a)	I am not interested in/do not like visiting nature areas	false	true
b)	My health does not allow me to visit nature areas	false	true
c)	There are no suitable nature areas within walking distance	false	true
d)	I feel insecure in nature areas	false	true
e)	I do not have time to visit nature areas	false	true
f)	I have a garden and that's enough	false	true

2.3 Reasons to visit nature areas

18. What was your reason to visit nature areas before the Covid-19 pandemic? (Only those who responded with alternative 1, 2 or 3 to question 15)

22. What was your reason to visit nature areas during the Covid-19 pandemic? (Only those who responded with alternative 1, 2 or 3 to question 20)

Response alternatives:

	**1**	**2**	**3**	**4**	**5**
I visited natural areas…	never	seldom	sometimes	quite often	very often
**a** … To be in the fresh air					
**b** … To recover from stress					
**c** … For physical activity					
**d** … To experience silence/nature sounds					
**e** … For social reasons					
**f** … To see other people					
**g** … To walk my dog (or other pet)					
**h** … To relax					
**i** … To enjoy the beauty of nature					
**j** … To be alone					
**k** … For spiritual experiences					
**l** … Because somebody else told me to do that					
**m** … Because it is good for my health					
**n** … To clear my head/think clearly					
**o** … Because my work requires it					
**p** … Because it's part of my regular transportation route					
**r** Because of other reasons					

2.4 What kind of natural areas did you visit?

19. What kind of natural areas did you visit before the Covid-19 pandemic and how often?

23. What kind of natural areas did you visit during the Covid-19 pandemic and how often?

Response alternatives:

	**1**	**2**	**3**	**4**	**5**
	never	seldom	sometimes	quite often	very often
**a** Private garden					
**b** Park					
**c** Forest					
**d** Freshwater bodies of water					
**e** Saltwater beach/boating					
**f** Nature reserve					
**g** Green play parks					
**h** Other type of area					

3. Alcohol consumption

"Alcohol" includes medium or strong beer, alcoholic cider, wine, and spirits
One standard glass corresponds to 12 g of alcohol. This amount of alcohol is found in a glass (15 cl) of wine, a bottle (33 cl) of strong beer (5%) or 4 cl of spirits.
28. How often did you use to drink alcohol before the Covid-19 pandemic?

31. How often are you drinking alcohol during the Covid-19 pandemic?

Response alternatives:

**1**	**2**	**3**	**4**	**5**
≥4 times/week	2-3 times/week	2-4 times/month	≤1 times/month	never

29. How many glass/day did you typically drink when you drank alcohol before the Covid-19 pandemic?

32. How many glass of alcohol/day do you typically drink during the Covid-19 pandemic?

Response alternatives:

**1**	**2**	**3**	**4**	**5**	**6**
1-2	3-4	5-6	7-9	10 or more	I do not know

4. Physical inactivity

25. The last question (referring to the IPAQ short form) is about the time you spent sitting on weekdays during a regular week pre-COVID. Include time spent at work, at home, while doing course work and during leisure time. This may include time spent sitting at a desk, visiting friends, reading, or sitting or lying down to watch television.

Before the Covid-19 pandemic, how much time did you usually spend sitting on one of days on a regular week?

27. The last question (referring to the IPAQ short form) is about the time you spent sitting on weekdays during a regular week during the Covid-19 pandemic. Include time spent at work, at home, while doing course work and during leisure time. This may include time spent sitting at a desk, visiting friends, reading, or sitting or lying down to watch television.

During the Covid-19 pandemic, how much time did you usually spend sitting on one of days on a regular week?

5. Mental Health

5.1 Perceived Stress Scale- PSS

33. The questions in this scale ask you about your feelings and thoughts during the last month. In each case, you will be asked to indicate by circling how often you felt or thought a certain way.

	**1**	**2**	**3**	**4**	**5**
In the last month, how often have you...	Never	Almost never	Sometimes	Fairly often	Very often
**a** … been upset because of something that happened unexpectedly?	0	1	2	3	4
***b** … felt that you were unable to control the important things in your life?	0	1	2	3	4
**c** … felt nervous and “stressed”?	0	1	2	3	4
***d** …felt confident about your ability to handle your personal problems in the last month?	4	3	2	1	0
***e** … felt that things were going your way?	4	3	2	1	0
***f** … felt that difficulties were piling up so high that you could not overcome them?	0	1	2	3	4

* the questions included in the classic four-item short scale

5.2 RAND-36: mental health/wellbeing and vitality scores

34. These questions are about how you feel and how things have been with you during the last four weeks. For each question, please give the answer that comes closest to the way you have been feeling. RAND-36: mental health/wellbeing and vitality scores

	**1**	**2**	**3**	**4**	**5**	**6**
How much of the time in the last month did you feel …	All of the time	Most of the time	A good bit of the time	Some of the time	little bit of the time	None of the time
****a** … full of pep?	100	80	60	40	20	0
***b** … as a very nervous person?	0	20	40	60	80	100
***c** … so down in the dumps that nothing could cheer you up?	0	20	40	60	80	100
***d** … calm and peaceful?	100	80	60	40	20	0
****e** … a lot of energy?	100	80	60	40	20	0

***f** … downhearted and blue?	0	20	40	60	80	100
****g** … worn out?	0	20	40	60	80	100
***h** … as a happy person?	100	80	60	40	20	0
****i** … tired?	0	20	40	60	80	100

* the questions included in the mental health/wellbeing subscale

** the questions included in the vitality subscale

5.3 (Copenhagen Psychosocial Questionnaire COPSOQ)

35. These questions are about how you feel and how things have been with you during the last month. For each question, please give the answer that comes closest to the way you have been feeling.

	**1**	**2**	**3**	**4**	**5**
How much of the time during the past month have you...	All the time	A large part of the time	Part of the time	A small part of the time	Not at all
**a** … had problems concentrating?	100	75	50	25	0
**b** … had difficulty in taking decisions?	100	75	50	25	0
**c** … had difficulty with remembering?	100	75	50	25	0
**d** … found it difficult to think clearly?	100	75	50	25	0

5.4 SLC90: depression and anxiety scores

36. How much were you during the last week bothered by…

	**1**	**2**	**3**	**4**	**5**
How much were you during the last week bothered by…	Not at all	A little bit	Moderately	Quite a bit	Extremely
***a** … feeling low in energy or slowed down	0	1	2	3	4
***b** … feeling blue	0	1	2	3	4
***c** … blaming yourself for things	0	1	2	3	4
***d** … worrying too much about things	0	1	2	3	4
***e** … feeling no interest in things	0	1	2	3	4
***f** … feeling everything is an effort	0	1	2	3	4
****g** … nervousness or shakiness inside	0	1	2	3	4
****h** … spells of terror or panic	0	1	2	3	4

* the questions included in the depression subscale

** the questions included in the anxiety subscale

6. Walkability

When we talk about "your neighbourhood", we mean the area within about 10 minutes walking distance around your home.

"Major streets" refers to roads that connect different districts and are used for e.g. public transport and transportation of goods.

"Minor streets" refers to roads that are mostly used to reach a specific address and that are rarely used by larger vehicles or public transport.

45. Think about the sidewalks/bike roads or other paved paths in your neighbourhood……

Supplementary material

	1	2	3	4
Think about the sidewalks/bike roads or other paved paths in your neighbourhood...	always/usually	half the time	seldom/never	(N/A)
a Are there paved sidewalks/bike paths along the major streets in your neighbourhood?				
b Are there paved sidewalks/bike paths along the minor streets in your neighbourhood?				
c Are the sidewalks/paths in good repair, without areas of uneven or broken pavement?				
d Are the sidewalks/paths lighted for use at night?				
e Are the sidewalks/paths wide enough for at least two adults to walk side by side?				
f Are there curbs to separate the sidewalk/paths from the street?				
g Are there curbs to separate the sidewalk/paths from the street?				
h Could someone use the sidewalk/paths using a wheelchair, walker, stroller, or other mobility aide without difficulty?				
i Are the sidewalk/paths free of items that might block free walking such as fire hydrants, light poles, signs, etc. in?				
j Are the sidewalks/paths and the area next to them clear of weeds, brush, broken glass and unsightly garbage?				
k Are there any benches or other places to rest along your sidewalk/paths?				
l Do any of the sidewalks or bike roads connect to major streets or other neighbourhoods?				

46. Think about major intersections in your neighbourhood...

	**1**	**2**	**3**
	yes	no	no major intersections
**a** Are there marked crosswalks at major intersections in your neighbourhood?			
**b** Are there functional crossing signals at major intersections in your neighbourhood?			

48. Think about the places that are within a 10 minute walk of your home. Are these destinations connected to your home by sidewalks or paved paths? In other words, would it be possible to get there by walking on a sidewalk or paved path?

49. IF YES, is this destination connected to your home by sidewalks or paved paths? 0=N/A means that this destination was not in walking distance (previous questions)

Is this destination within a 10 minute walk of your home?			IF YES, is this destination connected to your home by sidewalks or paved paths?			
	1	2		1	2	3

Supplementary material

48.	yes	no	***49.***	mostly	about half of the road	only a small part of the road
a grocery store/supermarket			***a***			
b place of worship			***b***			
c community centre			***c***			
d school or childcare facility			***d***			
e park or recreational facility			***e***			
f restaurant or other places to eat			***f***			
g retail store or other shopping			***g***			
var_48h personal services (hair care, nail salon, dry cleaners, laundry, etc.)			***var_49h***			
var_48i post office			***var_49i***			
var_48j cash machine			***var_49j***			
var_48k health care centre			***var_49k***			
var_48l bus stop			***var_49l***			

Part C – Supplementary results

Table S1.C: Between group differences in the percentage of people visiting natural areas "often" pre-pandemic versus during the pandemic.

Variable	Subgroups compared	Mean (95 % CI)
Age	*< 70 years - ≥ 70 years*	5.95 (1.5;10.41)*
Sex	*men - women*	-2.49 (-5.96;0.99)
Education	*basic – high school*	-7.14 (-15.21;0.93)
	high school – university	-3.59 (-7.56;0.37)
	basic – university	-10.73 (-18.38;-3.08)*
Ethnicity	*not born in SSB - born in SSB*	-5.83 (-11.43;-0.24)*
	neither mother nor father from SSB - both or one of the parents from SSB	-5.27 (-10.6;0.07)
Walkability	*Poor – Good*	2.56 (-1.83;6.96)
	Good – Very Good	1.62 (-2.54;5.78)
	Poor – Very Good	4.18 (-0.09;8.45)
NDVI in 50m buffers	*Low – Average*	-2.16 (-6.49;2.17)
	Average – High	0.02 (-4.12;4.16)
	Low – High	-2.14 (-6.39;2.11)

Table S2.C: What was your reason, before/during the Covid-19 pandemic, not to visit nature areas more often?

	beofore Covid-19	during Covid-19	difference between before and during Covid -19			
n=67	%	%	%	95 % CI		p
I am not interested in/do not like visiting nature areas	49.3	44.8	4.5	-3.3	12.3	0.2599
My health does not allow me to visit nature areas	6.0	4.5	1.0	-1.5	4.5	0.3210
There are no suitable nature areas within walking distance	16.4	17.9	1.5	-3.7	6.7	0.5676
I feel insecure in nature areas	3.0	6.0	3.0	-1.1	7.2	0.1589
I do not have time to visit nature areas	23.9	23.9	0.0	-8.5	8.5	1.0000
I have a garden and that's enough	19.4	20.9	1.5	-1.5	4.5	0.3210

Table S3.C: NDVI within 50m buffers and the average Mental Health scores, stratified by walkability.

	Walkability below median					Walkability above median				
		NDVI low		NDVI high			NDVI low		NDVI high	
Income low	N		N		p	N		N		p
Mental health score (RAND36)	436	68,817 (67,002;70,631)	616	71,805 (70,38;73,231)	0,01*	580	74,021 (72,713;75,328)	394	74,863 (73,31;76,416)	0,417
Vitality score (RAND36)	436	56,709 (54,711;58,706)	616	59,976 (58,339;61,613)	0,013*	580	62,526 (60,939;64,113)	394	62,716 (60,756;64,676)	0,882
Anxiety score (SCL90)	436	1,466 (1,291;1,64)	617	1,246 (1,119;1,373)	0,041*	580	1,059 (0,938;1,18)	395	0,922 (0,797;1,046)	0,133
Depression score (SCL90)	437	6,915 (6,372;7,458)	617	5,976 (5,567;6,384)	0,006*	580	5,16 (4,769;5,552)	395	5,273 (4,824;5,723)	0,712

Supplementary material

Perceived Stress Scale (PSS)	431	9,376 (8,94;9,812)	607	8,697 (8,371;9,023)	0,013*	575	8,245 (7,923;8,567)	391	8,136 (7,753;8,518)	0,668
The Cognitive Stress Score (COPSOQ)	437	33,724 (31,679;35,769)	617	31,118 (29,472;32,765)	0,05*	579	29,911 (28,309;31,514)	397	29,943 (27,928;31,959)	0,981

Table S4.C: Linear regression models adjusted for sex and age only

	**Buffer size**		
	**50m**	**300m**	**500m**
Mental health score (RAND36)			
NDVI	1.611 (3.535;6.756)	0.606 (-5.313;6.526)	-0.908 (-7.102;5.286)
Sex	-3.141 (-4.615;-1.667)*	-3.15 (-4.623;-1.676)*	-3.155 (-4.629;-1.682)*
Age	0.271 (0.224;0.317)*	0.271 (0.224;0.318)*	0.271 (0.224;0.317)*
Vitality score (RAND36)			
NDVI	0.67 (-5.312;6.651)	-2.047 (-8.927;4.833)	-2.941 (-10.139;4.258)
Sex	-2.754 (-4.467;-1.041)*	-2.767 (-4.48;-1.054)*	-2.769 (-4.481;-1.056)*
Age	0.354 (0.299;0.408)*	0.353 (0.299;0.408)*	0.353 (0.299;0.407)*
Anxiety score (SCL90)			
NDVI	0.151 (0.615;0.314)	0.047 (-0.487;0.582)	0.078 (-0.482;0.637)
Sex	0.228 (0.095;0.361)*	0.229 (0.096;0.363)*	0.229 (0.096;0.363)*
Age	-0.026 (-0.03;-0.021)*	-0.026 (-0.03;-0.021)*	-0.026 (-0.03;-0.021)*
Depression score (SCL90)			
NDVI	0.221 (-1.302;1.743)	0.243 (-1.509;1.994)	0.583 (-1.249;2.416)
Sex	0.69 (0.254;1.126)*	0.69 (0.254;1.126)*	0.691 (0.255;1.127)*
Age	-0.073 (-0.087;-0.06)*	-0.073 (-0.087;-0.06)*	-0.073 (-0.087;-0.059)*
Perceived Stress Scale (PSS)			
NDVI	0.31 (1.53;0.91)	-0.323 (-1.727;1.081)	-0.2 (-1.67;1.27)
Sex	0.708 (0.358;1.058)*	0.709 (0.359;1.059)*	0.71 (0.36;1.059)*
Age	-0.071 (-0.082;-0.06)*	-0.071 (-0.082;-0.06)*	-0.071 (-0.082;-0.06)*
The Cognitive Stress Score (COPSOQ)			
NDVI	-1.476 (-7.578;4.625)	-2.894 (-9.915;4.127)	-2.338 (-9.688;5.012)
Sex	3.649 (1.901;5.397)*	3.647 (1.899;5.394)*	3.651 (1.904;5.399)*
Age	-0.328 (-0.383;-0.273)*	-0.328 (-0.384;-0.273)*	-0.329 (-0.384;-0.273)*

Table S5.C: Mental health estimates associated with NDVI within different buffer sizes (models adjusted as in table 6). (*p<0.05; CI – Confidence Interval)

**Mental health estimate**	**100m**	**300m**	**500m**
Mental health score (RAND36)	6,017 (0,514;11,521)*	6,563 (0,531;12,594)*	5,521 (-0,8;11,842)
Vitality score (RAND36)	4,494 (-1,844;10,832)	6,563 (0,531;12,594)*	4,096 (-3,182;11,375)
Anxiety score (SCL90)	-0,598 (-1,1;-0,096)*	-0,492 (-1,042;0,058)	-0,502 (-1,078;0,074)
Depression score (SCL90)	-1,303 (-2,932;0,327)	-1,627 (-3,413;0,158)	-1,596 (-3,114;-0,079)*
Perceived Stress Scale (PSS)	-1,165 (-2,485;0,156)	-1,553 (-3,001;-0,106)*	-1,596 (-3,114;-0,079)*
The Cognitive Stress Score (COPSOQ)	-6,802 (-13,389;-0,215)*	-9,029 (-16,245;-1,812)*	-9,379 (-16,942;-1,816)*

Supplementary material

Table S6.C: Fully adjusted linear spline models with the knot set to NDVI = 0.5 (*p<0.05; CI – Confidence Interval)

		50m	100m	300m	500m
Mental health score (RAND36)	below 0.5	5.418 (-1.571;12.408)	4.301 (-3.041;11.642)	1.589 (-6.423;9.601)	-1.717 (-10.119;6.685)
	above 0.5	8.426 (-13.601;30.453)	14.301 (-9.778;38.379)	29.602 (4.422;54.781)*	39.867 (12.806;66.927)*
Vitality score (RAND36)	below 0.5	5.386 (-2.664;13.436)	3.916 (-4.541;12.372)	3.246 (-5.991;12.482)	-1.955 (-11.637;7.726)
	above 0.5	2.312 (-23.055;27.679)	7.285 (-20.449;35.019)	8.661 (-20.366;37.689)	32.816 (1.633;63.999)*
Anxiety score (SCL90)	below 0.5	-0.367 (-1.004;0.27)	-0.212 (-0.88;0.457)	-0.057 (-0.788;0.673)	0.106 (-0.661;0.872)
	above 0.5	-1.77 (-3.777;0.238)	-2.461 (-4.654;-0.268)*	-2.504 (-4.794;-0.214)*	-3.38 (-5.845;-0.915)*
Depression score (SCL90)	below 0.5	-1.074 (-3.144;0.996)	-0.224 (-2.397;1.95)	-0.028 (-2.399;2.342)	0.806 (-1.681;3.293)
	above 0.5	-2.523 (-9.047;4.001)	-6.506 (-13.631;0.619)	-9.017 (-16.448;-1.586)*	-11.967 (-19.966;-3.969)*
Perceived Stress Scale (PSS)	below 0.5	-1.389 (-3.071;0.293)	-1.308 (-3.073;0.457)	-1.012 (-2.937;0.912)	-0.486 (-2.504;1.532)
	above 0.5	-0.332 (-5.646;4.982)	-0.477 (-6.263;5.31)	-4.059 (-10.109;1.991)	-6.88 (-13.389;-0.371)*
The Cognitive Stress Score (COPSOQ)	below 0.5	-7.857 (-16.229;0.515)	-8.611 (-17.405;0.183)	-9.649 (-19.241;-0.057)*	-7.004 (-17.07;3.061)
	above 0.5	0.876 (-25.443;27.196)	1.912 (-26.906;30.73)	-6.159 (-36.246;23.929)	-20.66 (-53.103;11.783)
